# Identification and Functional Verification of Cold Tolerance Genes in Spring Maize Seedlings Based on a Genome-Wide Association Study and Quantitative Trait Locus Mapping

**DOI:** 10.3389/fpls.2021.776972

**Published:** 2021-12-09

**Authors:** Yukun Jin, Zhongren Zhang, Yongjing Xi, Zhou Yang, Zhifeng Xiao, Shuyan Guan, Jing Qu, Piwu Wang, Rengui Zhao

**Affiliations:** ^1^Joint Laboratory of International Cooperation in Modern Agriculture Technology of Ministry of Education, Plant Biotechnology Center, Jilin Agricultural University, Changchun, China; ^2^Novogene Bioinformatics Institute, Novogene Co., Ltd, Beijing, China

**Keywords:** cold tolerance, GWAS, QTL mapping, gene cloning, functional annotation

## Abstract

Maize (*Zea mays* L.) is a tropical crop, and low temperature has become one of the main abiotic stresses for maize growth and development, affecting many maize growth processes. The main area of maize production in China, Jilin province, often suffers from varying degrees of cold damage in spring, which seriously affects the quality and yield of maize. In the face of global climate change and food security concerns, discovering cold tolerance genes, developing cold tolerance molecular markers, and creating cold-tolerant germplasm have become urgent for improving maize resilience against these conditions and obtaining an increase in overall yield. In this study, whole-genome sequencing and genotyping by sequencing were used to perform genome-wide association analysis (GWAS) and quantitative trait locus (QTL) mapping of the two populations, respectively. Overall, four single-nucleotide polymorphisms (SNPs) and 12 QTLs were found to be significantly associated with cold tolerance. Through joint analysis, an intersection of GWAS and QTL mapping was found on chromosome 3, on which the *Zm00001d002729* gene was identified as a potential factor in cold tolerance. We verified the function of this target gene through overexpression, suppression of expression, and genetic transformation into maize. We found that *Zm00001d002729* overexpression resulted in better cold tolerance in this crop. The identification of genes associated with cold tolerance contributes to the clarification of the underlying mechanism of this trait in maize and provides a foundation for the adaptation of maize to colder environments in the future, to ensure food security.

## Introduction

Various abiotic stresses, such as low temperature, drought, and high salinity, significantly affect the normal growth and yield of plants. Of these, cold stress has had an impact on the growth, yield, and spatial distribution of crops, including maize (*Zea mays* L.). Cold tolerance in maize is a complex quantitative hereditary trait, controlled by multiple genes; different periods of this crop are controlled by different genetic mechanisms, which are easily affected by environmental factors (Hodges et al., 1997; Gao et al., 2009). Jilin Province is one of the most important sites of maize cultivation in China; however, it is extremely prone to cold damage, resulting in reduced maize production (Jena et al., 2012; Ma et al., 2018; Yan et al., 2018).

Several reports identifying molecular markers and quantitative trait locus (QTLs) associated with cold tolerance have been published, but their practical application in maize breeding to improve resistance to cold conditions has so far been limited (Revilla et al., 2014). Many studies have shown that cold tolerance at the germination and seedling stages of maize is a quantitative trait, controlled by multiple genes (Trzcinska-Danielewicz et al., 2009); studies have also shown that epistasis and additive and dominant gene effects in maize germination under low temperature significantly affect tolerance (Ma et al., 2007).

Rodríguez et al. (2008) used a recombinant inbred line (RIL) constructed using a cross between the inbred lines B73 × Mo17 to locate QTLs affecting maize leaf color under low temperatures; they found that the major QTL that control maize leaf color under these conditions is located at the chromosome positions bin 3.01 and bin 6.03. Jompuk et al. (2005) used the F_2_ population ETH-DH7 × ETH-DL3 to identify the main QTL governing the operation of the photosynthetic system at low temperatures during the seedling stage of maize, which was found to be distributed along chromosome 6. Leipner et al. (2008) also used this F_2_ population ETH-DH7 × ETH-DL3 to assess cold tolerance in the maize seedling photosynthetic system under low temperature stress and detected that the major QTL for this trait was distributed along chromosome 3. Pimentel et al. (2005) used the constructed F_2_ population W6786 × IL731A to identify the QTL controlling the degree of photoinhibition in maize under low temperature stress; three QTLs on chromosomes 3, 4, and 8 were detected in plants cultivated under controlled indoor and outdoor conditions. The QTL in bin 3.09 on chromosome 3 was detected in both indoor and outdoor plants; the other two QTLs were only detected in indoor environments. Li et al. (2018) used two cold-tolerant inbred lines, 220 and P9-10, and two cold-sensitive inbred lines, Y1518 and PH4CV, to construct three F_2:3_ populations; and using the germination rate as the phenotype to QTL mapping, they identified 43 QTLs that each explained 0.62–39.44% of the phenotypic variation among plants in this population. Seven of these QTLs together explained more than 10% of the total phenotypic variation. Hu et al. (2016) identified a total of six QTLs governing seed germination under low temperature conditions, on chromosomes 4, 5, 6, 7, and 9, under different degrees of cold stress, which were found to explain 3.39–11.29% of phenotypic variation.

Strigens et al. (2013) conducted a genome-wide association study (GWAS) in maize, locating 19 single-nucleotide polymorphisms (SNPs) related to cold tolerance, which explained 5.7–52.5% of the phenotypic genetic variation in maize seedlings. Meanwhile, a number of candidate cold tolerance genes were discovered in the Strigens’ study. Hund et al. (2004) used the F_2:3_ population, Lo964 × Lo1016, to identify QTL controlling cold-related traits of maize seedlings under cold stress. A major QTL was found on chromosome 5 that could explain 14% of the total phenotypic variation of root diameters. Fracheboud et al. (2002, 2004) chlorophyll fluorescence parameters, CO2 exchange rate, leaf greenness, shoot dry matter, and shoot nitrogen content as phenotype in an F_2:3_ population under cold stress and found that a major QTL located on chromosome 6 explained 37.4% of the phenotypic variance in the chronic photoinhibition at low temperature. Rodríguez et al. (2014) used the F_2:3_ population, EP42 and A661, to map QTLs using four cold tolerance-related traits, number of survival plants, dry weight, quantum yield of photosystem II, and total anthocyanin content. Four QTLs associated with cold stress were identified three genomic regions in chromosomes 2, 4, and 8.

In our study, we used GWAS and QTL mapping to identify major SNPs and QTL in two populations of maize. We used an integrative analysis approach, combining the results of the GWAS and QTL mapping, to further identify key candidate genes for cold tolerance. We then performed a functional annotation and cloning into *Z. mays* for functional verification of these candidate genes. Using these methods, we aimed to provide the basis for developing possible strategies for breeding new maize with improved cold tolerance, thus improving food security.

## Materials and Methods

### Plant Materials and Experimental Design

There were two genetic populations in our study: the first (population 1) was a natural population consisting of 80 backbone inbred lines. These lines were selected from numerous maize materials based on their frequency and genetic diversity in maize breeding in Jilin Province, China (Supplementary File 1). The second population (population 2) was an F_2_ population of 210 offspring, with parents derived from population 1 (W72 × W10). Two lines W10 (chilling-tolerant) and W72 (chilling-sensitive) were used as parents to produce a segregating F_2_ population. To obtain F_2_ hybrids, we self-crossed F_1_ individuals, and then self-crossed F_2_ individuals to obtain F_2:3_ seeds. The above experimental materials were provided by the Biotechnology Center of Jilin Agricultural University, China.

### Analysis of Phenotypic Data and Peroxidase Activity

We measured the peroxidase (POD) activity of each maize inbred line using the guaiacol method (Javadian et al., 2010) at seedling stage. Three consecutive batches of tests were performed on each inbred line at a low temperature of 6°C for 24 h. Ten strains of the same line were tested in each test, and average values of all 30 strains were obtained for subsequent analysis, which was conducted using SPSS 23.0 software (Pallant, 2013).

The formula for calculating POD activity was as:


$$\mathrm{POD}\;\left(U/g\times \min\right)=\frac{A470•\mathrm{Vt}}{0.01\times W•\mathrm{Vs}•t}$$

where A470 was the change in absorbance within the reaction time; *W* was the fresh weight (g); *Vt* was the total volume of extracted enzyme solution (ml); *Vs* was the volume of enzyme solution during measurement (ml); and *t* was time (min).

### DNA Extraction for Sequencing in Populations 1 and 2

The leaves of populations 1 and 2 were collected and flash frozen in liquid nitrogen. The DNA was extracted using the NuClean Plant Genomic DNA Kit (CWBio, Jiangsu, China); the concentration and purity of the extracted DNA were determined using a Nano Drop 2000 micro spectrophotometer (Thermo Fisher, Waltham, MA, United States). The integrity of the extracted DNA was confirmed using agarose gel electrophoresis (agarose concentration 1%, voltage 200 V, time 25 min; Zhao et al., 2018).

### Whole-Genome Sequencing and SNP Identification in Population 1

We used the NuClean Plant Genomic DNA Kit (CWBio, Jiangsu, China) to extract genomic DNA from the 80 inbred maize lines in population 1 at seedling stage. DNA quality was assessed using 1% agarose gel electrophoresis under 150 volts 70 milliampere, 30 min and a NanoDrop 2000 spectrophotometer (NanoDrop, Wilmington, DE, United States). Qualified DNA samples with clear bands were used to construct a genomic library. Sequencing was performed using an Illumina HiSeq PE150 (Illumina Inc., San Diego, CA, United States) to obtain raw data. Then, read pairs containing the linker sequence were filtered out; paired reads with N content in a single-end sequencing read exceeding 10% of the length of the read, and those with a number of low-quality bases (defined as quality value Q ≤ 5) in a single-end sequencing read exceeding 50% of the length of the read, were removed. Finally, the sequencing data were left high-quality, clean genomic data.

Maize B73 RefGen_v4 was the data mining resource of the Maize Genetics and Genome Database (MaizeGDB).^1^ We compared these clean data to the reference genome, Maize B73 RefGen_v4 (Shamimuzzaman et al., 2020), using the Burrows-Wheeler Aligner (BWA) software (Jia et al., 2013), and SAMtools software (Zhou et al., 2015) was used to remove duplicates (command: rmdup). Then, we used a Bayesian model to detect polymorphic sites in the population, and filtered and screened high-quality SNPs using a minor allele frequency (MAF) > 0.05 and call rate >80%. Finally, we used ANNOVAR software (Yang and Wang, 2015) to functionally annotate the identified SNPs.

### Population Structure, Linkage Disequilibrium, GWAS, and Candidate Gene Annotation in Population 1

The phylogenetic tree was inferred using TreeBeST^2^ using the *p*-distances genetic distance estimation model and the identified SNPs. The Principal Component Analysis (PCA) was analyzed by Genome-wide Complex Trait Analysis (GCTA; Yang et al., 2011).^3^

We used Haploview (Barrett et al., 2005) to calculate linkage disequilibrium (LD) in population 1 and FarmCPU to perform a GWAS to identify associations between genetic loci and phenotypic traits (Kaler et al., 2020). Research in recent years had shown that the SNPs at which the maximum value of *R*^2^ (corresponding to the LD) decays to half are linked. When *R*^2^ decayed to half, the corresponding LD was about 5.0 kb. Therefore, we chose a LD of 5.0 kb to scan for functional genes.

All identified functional genes were annotated using the SWISS-PROT,^4^ COG,^5^ GO,^6^ KEGG,^7^ and NCBI^8^ databases; possible candidate cold tolerance genes were screened based on previous studies.

### Genotyping by Sequencing in Population 2

Given that the parental line of population 2 had already been sequenced among population 1, we used GBS to sequence population 2 only. We used qualified samples with clear bands in electrophoresis for GBS and quality analysis of SNPs. Sequencing was completed using Illumina NovaSeq 6,000 (Illumina Inc., San Diego, CA, United States).

The GBS data were filtered to obtain clean data, using the same criteria as for population 1, detailed in section “Whole-Genome Sequencing and SNP Identification in Population 1.”

We used BWA software (Jia et al., 2013) to compare the clean data with the reference genome (Maize B73 RefGen_v4) and used SAMtools to convert the compared result format into SAM/BAM files; in addition, the Perl script was used to calculate the mapping rate and coverage, and SAMtools “sort” command was used to detect mutations. Polymorphic tags of parental line were genotyped to facilitate subsequent genetic analysis. Because the parents were homozygous inbred lines with aa and bb genotypes, SNPs with the segregating pattern of aa × bb were screened (Zhang et al., 2016a). Three standards were used for the screening of SNP: first, in the offspring typing, there may be a few base types that did not appear in the parents, and we considered them as deletions; second, included genotypes covered at least 80% of SNPs in the offspring; and third, segregation distortion. We used the chi-square test to assess offspring with a significance threshold of *p* < 0.001.

### Construction and Evaluation of the Genetic Linkage Map

We used Join Map 4.1(Stam, 1993) software to construct a genetic linkage map of high-quality SNPs obtained after screening. First, the linkage group was divided into chromosomes; second, a maximum likelihood method was used to sort the markers in each linkage group. That was, A and B were linked, B and C were linked, and then, A and C were linked; otherwise, B and C were not linked, then A and C were not linked. Third, the genetic distance between markers was calculated using Kosambi mapping function. Finally, we used the Perl SVG module to draw the genetic linkage map.

To ensure the quality of the genetic linkage map, we created a map of the haploid source of population 2 in 10 linkage groups, and possible double crossover sites were identified; we also evaluated linkage relationships between each of the markers in the same linkage group on the map (Zhao et al., 2016); and finally, we performed a collinearity analysis by the R programming language using marker positions on the genome and the genetic map to perform collinearity analysis (Zhang et al., 2016b).

### Screening QTLs Associated With POD

QTLs were detected using composite interval mapping (CIM) in WinQTL Cart 2.5 software (Jiang et al., 2018) utilizing the phenotype and genotype data from population 2. The Kosambi function was used with the scan step set as 1 cM, and the maximum value of *p* for entering variables in a stepwise regression of residual phenotype on marker variables (PIN) was set as 0.001. LOD (=0.217 likelihood ratio) threshold for declaring a putative QTL for each trait, data set, and model was defined by 1,000 permutations. Finally, we selected a QTL with a threshold logarithm of the odds (LOD) > 2.5 (Meng et al., 2015). Fracheboud et al. (2002) found the presence of a QTL was declared significant if the LOD value was >2.5 for a single trait analysis.

### Integrated GWAS and QTL Mapping Analysis

We integrated the results of the GWAS and QTL mapping in populations 1 and 2 to identify SNPs that were significantly associated with the POD phenotype. We used an LD of 5.0 kb to scan for functional genes.

### Expression Analysis of the Candidate Cold Tolerance Gene *Zm00001d002729*

We selected 10 strains in each of population 1 and 2 for expression analysis of the candidate gene *Zm00001d002729*; in population 1, we selected the extremely cold-resistant strains W10, W18, and W26; general cold-resistant strains W1, W15, W36, and W52; and extremely cold-sensitive strains W43, W48, and W72. In population 2, we selected extremely cold-resistant strains J15, J75, and J144; general cold-resistant strains J8, J98, J183, and J191; and extremely cold-sensitive strains J107, J123, and J200. We extracted RNA from leaves of each inbred line as a template at seedling stage and measured the relative expression of *Zm00001d002729* in each line using quantitative real-time PCR (qRT-PCR) and the fluorescent dye SYBR Green (Takara Biomedical Technology Co., Ltd., China). The internal reference gene we chose was *Actin-1* (*Act*; Accession: J01238). The primers used are given in Supplementary File 2.

Total RNA was extracted using the Plant RNA Kit R6827-02 (Omega Bio-tek, United States), and then reverse-transcribed into cDNA using the All-in One™ First-Strand cDNA Synthesis Kit (GeneCopoeia Inc., United States); qRT-PCR primers were designed using an online tool (https://sg.idtdna.com/PrimerQuest/Home/Index). qRT-PCR was performed using the All-in One™ qPCR Mix (GeneCopoeia Inc., United States) and the Stratagene Mx3000P PCR system (Agilent Technologies, United States) with three repetitions, and the relative expression level of the genes was calculated using the 2^-ΔΔCT^ method (Lu et al., 2018).

### Cloning of *Zm00001d002729* and Vector Construction

We used Primer Premier 5.0 (Wei et al., 2009) to design specific primers for cloning *Zm00001d002729* by RT-PCR, using cDNA from leaf tissue of maize strain Dan 340 at seedling stage as the template, and annealing temperature was about 55.0°C (Supplementary File 2). Then, we constructed the pMD-18T-*Zm00001d002729* cloning vector and sequenced it to confirm successful cloning. We used the Seamless Assembly Cloning Kit (Taihe Biotechnology Co., Ltd., China) to construct the pCAMBIA3301-*Zm00001d002729* expression vector. We used the CRISPR-P Web site^9^ to design small guide RNA (sgRNA). We selected sgRNA with high-target scores, low miss-target rates, and used Seamless cloning to construct a pCXB053-*Zm00001d002729*-CRISPR/Cas9 vector with a single gene and dual targets. Meanwhile, we used U6 as a promoter to construct a CRISPR/Cas9 gene editing vector.

### Genetic Transformation of *Zm00001d002729* Into *Z. mays* H299 and Functional Verification

In order to verify the function of Zm00001d002729, we transformed Zm00001d002729 into strain H299 using an Agrobacterium-mediated method (Frame et al., 2006). In this study, Agrobacterium-mediated methods were used to transfer the constructed expression vector to the recipient. After embryoculture, subculture, pre-culture, co-culture, screening culture, elongation culture, and rooting culture, we obtained T0 maize and cultivated it to the T2 generation. We performed test strip detection for *bar* gene by GeneTureTM BAR Test Kit (Artron Laboratories Inc.) and qRT-PCR detection on the transformed maize. Finally, we cultivated the T2 generation containing pCAMBIA3301-Zm00001d002729 or CRISPR/Cas9-Zm00001d002729, and three wild-type maize plants in total, to under conditions of cold stress at 6°C for 24 h at seedling stage, monitoring their morphology and measuring POD activity, malondialdehyde (MDA) content, and relative conductivity of seedlings leaves.

## Results

### Analysis of Phenotypic Data and POD Activity

**Figure 1:**
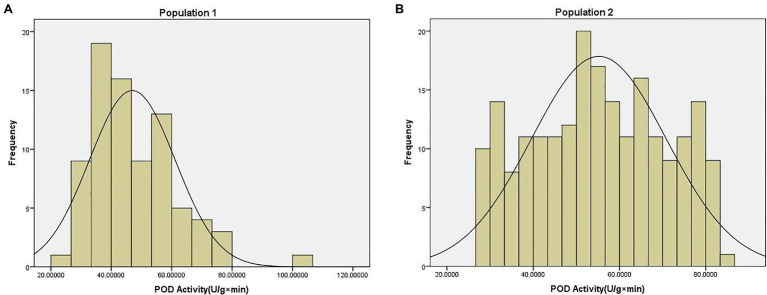
Phenotype distribution of peroxidase (POD) activity traits in **(A)** population 1 and **(B)** population 2.

The POD activity phenotype data of two population were shown in Supplementary File 3. The average POD activity in population 1 was 46.95 U/g × min, with a range of 22.97–100.75 U/g × min, and a coefficient of variation of 0.30. The average POD activity in population 2 was 55.19 U/g × min, with a range of 27.44–83.37 U/g × min, and a coefficient of variation of 0.28. These data were found to be normally distributed according to the Kolmogorov-Smirnov test (*p* = 0.20) of normality (Figure 1).

### Whole-Genome Sequencing and SNP Identification in Population 1

**Figure 2:**
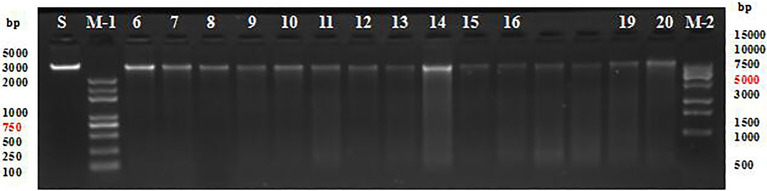
Agarose gel electrophoresis of DNA fragments from populations 1 and 2. S, standard sample; M-1, trans 5k DNA marker; M-2, trans 15k DNA marker; and 6–20, partial maize genome.

The quality of DNA extracted from the sample was high and the fragments were clear (Figure 2), thus meeting the requirements of DNA library construction for sequencing.

We performed WGS on population 1 and obtained 1.58 TB of clean data after quality control. Comparison with the maize inbred line reference genome showed that the mapping rate of the population samples was 98.72%. The average sequencing depth was 17.10×, and the average genome coverage was 89.16%. The similarity between each sample and the reference genome met the requirements of WGS analysis and had a very good sequencing depth and coverage. We obtained a total of 24,860,241 high-quality SNPs, of which 85.4% were intergenic. The results of SNP genotyping are shown in Supplementary File 4.

### Population Structure and Linkage Disequilibrium in Population 1

**Figure 3:**
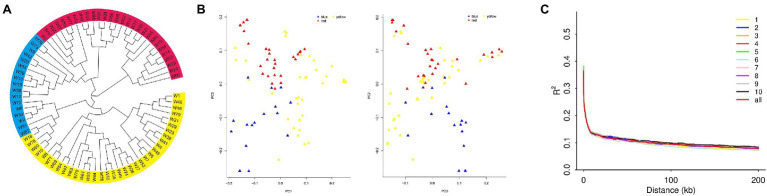
**(A)** The population structure, **(B)** Principal Component Analysis (PCA), and **(C)** linkage disequilibrium (LD) diagram for population 1.

We constructed a phylogenetic tree for population 1, based on genotype data of 80 inbred lines (Figure 3A). The results showed three subpopulations: subpopulation 1 (in red) contains lines mainly from Tang Si Ping Tou and Lv Da Red Bone; subpopulation 2 (in blue) contains lines mainly from Lancaster; and subpopulation 3 (in yellow) contains lines mainly from Reid. The PCA results were shown in Figure 3B and Supplementary File 5. The results showed the distributions of inbred lines, and they were separated accordingly.

The distribution of LD on chromosomes is described in the LD attenuation diagram (Figure 3C). The abscissa represents the distance at which LD occurs, and the ordinate is the correlation coefficient of LD, a way of displaying the LD value.

The attenuation distance was 3.7 kb in chromosome 1; 3.6 kb in chromosome 2; 3.5 kb in chromosome 3; 3.5 kb in chromosome 4; 3.1 kb in chromosome 5; 3.1 kb in chromosome 6; 2.6 kb in chromosome 7; 3.2 kb in chromosome 8; 3.8 kb in chromosome 9; and 3.2 kb in chromosome 10. The average attenuation distance of the whole genome was 3.4 kb.

### GWAS to Identify Genes Associated With POD

**Figure 4:**
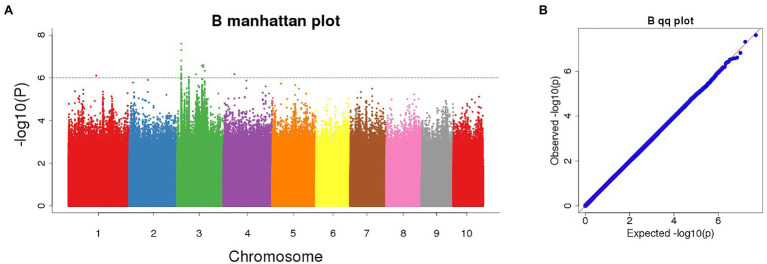
(A) Manhattan plot of significant SNPs correlating with POD activity using the FarmCPU model; (B) Quantile-Quantile (Q-Q) plot.

The Manhattan chart and Q-Q chart for the GWAS for POD activity are shown in Figure 4. We used *p* < 0.000001 as the threshold; our analysis detected four SNPs that were significantly related to POD activity. These SNPs were all located on chromosome 3 (Table 1).

**Table 1:** Single-nucleotide polymorphisms (SNPs) significantly associated with POD activity (*p* < 0.000001).

Name	Start (bp)	End (bp)	peakPOS (bp)	chr	ref	alt	peak_value	Candidate gene	LD
sPOD-20,592,562	20,587,562	20,597,562	20,592,562	3	A	G	6.10	*Zm00001d002729*	0
sPOD-96,084,381	96,079,381	96,089,381	96,084,381	3	C	T	6.17	*Zm00001d041082*	3,183
sPOD-133,504,984	133,499,956	133,509,984	133,504,984	3	T	A	6.60	*Zm00001d041691*	3,551
sPOD-140,877,969	140,872,969	140,882,969	14,087,7,969	3	T	C	6.33	*Zm00001d041853*	1,384

### GBS, Development, Encoding, and Screening of SNPs in Population 2

The two parents from population 1 (whose genomes were sequenced using WGS) and 210 offspring from population 2 (GBS) were successfully sequenced with an average sequencing depth of 30× and 10×, respectively. Our results yielded a total of 261.005 GB raw data from the offspring in population 2, with an average of 1,243 GB of raw data for each sample, and the filtered clean data was 260.879 GB, with 1.242 GB on average in each sample. The sequencing quality for the offspring was very high (Q20 ≥ 94% and Q30 ≥ 85%), and the sample GC distribution was normal. The database was successfully constructed and sequenced.

On comparison with the maize reference genome, 9,141,219 and 6,127,709 SNPs were identified in the parent plants, W72 and W10, respectively. A total of 5,759,868 SNPs were identified in population 2 compared with the reference genome, and 1,847,696 polymorphic SNPs were assigned to the parental aa × bb segregation pattern (Supplementary File 6). After filtering, 26,693 SNPs remained. We used these SNPs for genetic linkage map construction. SNPs were screened and adjusted iteratively to optimize the map following its initial construction.

### Construction and Evaluation of the Genetic Linkage Map

Each chromosome constituted a linkage group. A total of 8,213 SNPs were shown on the map, with a total genetic distance of 6516.35 cM. The average genetic distance between two adjacent markers was 0.79 cM. There were 1,163 markers in the first linkage group covering a genetic distance of 1114.99 cM: the longest genetic distance among the 10 linkage groups. On the other hand, there were 619 markers in the fifth linkage group over a genetic distance of 414.49 cM, covering the shortest genetic distance among the 10 linkage groups (Supplementary File 7). We also analyzed the distribution of gaps in each linkage group (Supplementary File 8); most gaps were less than 5 cM.

We used the Perl SVG module to draw a genetic linkage map (Supplementary File 9) and evaluated the quality of the map by constructing a map of the haploid source with all markers in each linkage group from population 2. The haploid source of the large intervals observed in every offspring was one of the parents, and the proportion of double crossing-over was less than 3%, indicating high quality of the linkage map (Supplementary File 9). We constructed a heat map of linkage relationships between each marker in the same linkage group from the genetic linkage map. The linkage relationship between adjacent markers in each group was very strong. With increasing distance, the linkage relationship between markers weakened gradually, indicating that the order of markers in the map was correct (Supplementary File 9). Finally, we analyzed the collinearity of the markers on the genome and the genetic map. The collinearity map can not only reflect the collinearity of the position of the markers on the genome and genetic map, but also reflect the distribution of the markers on the genetic map mapped to the chromosome in general. It can also reflect the relationship of each marker on the genome and genetic map. It can be seen from this figure that the distribution of markers on the chromosomes is relatively uniform, most of the marker sequences on each linkage group are basically consistent with the genome, the collinearity is good, and the calculation accuracy of the genetic recombination rate is high (Supplementary File 9).

### Screening of QTLs Associated With POD

**Figure 5:**
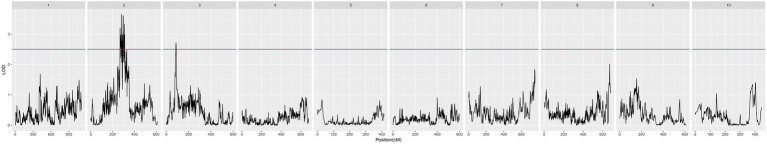
Quantitative trait locus (QTL) mapping of POD activity.

We detected a total of 12 QTLs related to POD. Among them, 11 QTLs were located on chromosome 2, and 1 QTL was located on chromosome 3. The QTL qPOD2b was located on chromosome 2, which had the largest contribution to POD activity of all QTLs, at 6.27%, with an additive effect of 52.80 U/g × min and a dominant effect of 6.51 U/g × min. qPOD3 was located on chromosome 3, with a contribution to the POD phenotype of 5.60%, an additive effect of 1.17, and a dominant effect of 4.52 (Figure 5; Table 2).

**Table 2:** Quantitative trait locus (QTLs) associated with POD activity.

Name	chr	Left marker	Right marker	Left position (bp)	Right position (bp)	LOD	Additive	Dominance	PVE (%)
qPOD2a	2	mk-2-411	mk-2-1,108	73,584,577	143,233,161	3.14	−57.66	6.30	5.41
qPOD2b	2	mk-2-1,107	mk-2-561	143,233,142	92,502,399	2.75	−52.80	6.51	6.27
qPOD2c	2	mk-2-561	mk-2-801	92,502,399	123,536,831	3.11	−55.91	6.18	5.63
qPOD2d	2	mk-2-801	mk-2-950	123,536,831	133,026,652	2.75	−58.22	6.45	5.47
qPOD2e	2	mk-2-972	mk-2-1,062	133,419,121	137,369,789	2.82	−119.36	5.84	5.94
qPOD2f	2	mk-2-947	mk-2-1,133	132,958,156	143,771,596	2.93	−119.36	5.80	5.62
qPOD2g	2	mk-2-1,133	mk-2-731	143,771,596	115,063,134	3.15	−119.36	6.19	5.77
qPOD2h	2	mk-2-731	mk-2-1,152	115,063,134	144,527,652	2.53	−119.36	5.89	5.90
qPOD2i	2	mk-2-1,157	mk-2-1113	144,789,539	143,268,023	2.97	−30.49	5.11	5.90
qPOD2j	2	mk-2-1,113	mk-2-998	143,268,023	133,848,401	2.81	−120.31	5.75	6.12
qPOD2k	2	mk-2-998	mk-2-1,174	133,848,401	146,341,832	2.77	−119.36	5.99	5.43
qPOD3	3	mk-3-75	mk-3-78	20,191,382	23,118,526	2.61	1.17	4.52	5.60

### Annotation and Screening of Cold Tolerance Candidate Genes in Populations 1 and 2

We integrated the results of the GWAS and QTL mapping in two populations to identify SNPs and QTLs that were significantly associated with the POD activity phenotype. Then, we used an LD of 5.0 kb to scan for functional genes. Our integrated GWAS and QTL mapping analysis revealed that the SNP (sPOD-20,592,562) was located in the QTL qPOD3 and that this SNP was associated with the gene *Zm00001d002729*. It meant that *Zm00001d002729* has been identified in both populations, which greatly increased the credibility of this gene. Our functional annotation indicated that this gene was an acyl-acyl carrier protein thioesterase.

We used Cell-PLoc 2.0^10^ to predict the subcellular localization of the Zm00001d002729 protein (Chou and Shen, 2008). The result showed that the predicted location was chloroplast and nuclear.

### Expression Analysis of Cold Tolerance Candidate Gene *Zm00001d002729*

**Figure 6:**
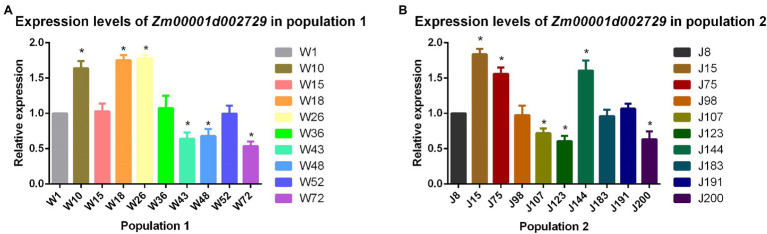
(A) Relative expression levels of *Zm00001d002729* in population 1 and (B) population 2.

We analyzed the relative expression levels of the candidate gene *Zm00001d002729* seedling stage in the two populations. The results showed that the expression level of this cold tolerance candidate gene in maize strains with better cold tolerance (W10, W18, W26, J15, J75, and J144) was significantly (*p* < 0.05) higher than that in cold-sensitive maize strains (W43, W48, W72, J107, J123, and J200). From this we concluded that higher expression levels of *Zm00001d002729* in maize result in greater cold tolerance (Figure 6).

### Cloning and Vector Construction of *Zm00001d002729*

We successfully obtained *Zm00001d002729* cDNA by RT-PCR method by using RNA extracted from the leaves seedling stage Dan 340 as the template. The full length of *Zm00001d002729* coding sequence was 690 bp.

After construction of the cloning vector, we used Seamless cloning technology to construct the plant over expression vector and CRISPR/Cas9 vector. Firstly, we used pCAMBIA3301 as the vector backbone and constructed pCAMBIA3301-*Zm00001d002729*. Meanwhile, we used U6 as a promoter to construct a CRISPR/Cas9 gene editing vector. Two carrier structures are shown in Supplementary File 10.

### Functional Verification of *Zm00001d002729*

We transformed *Zm00001d002729* into *Z. mays* H299 *via* Agrobacterium-mediated method; the growth process is shown in Supplementary File 11. After testing to confirm successful transformation using the GeneTure™ BAR Test Kit (Artron Laboratories Inc.; Supplementary File 12), we cultivated the genetically modified maize to the T2 generation.

**Figure 7:**
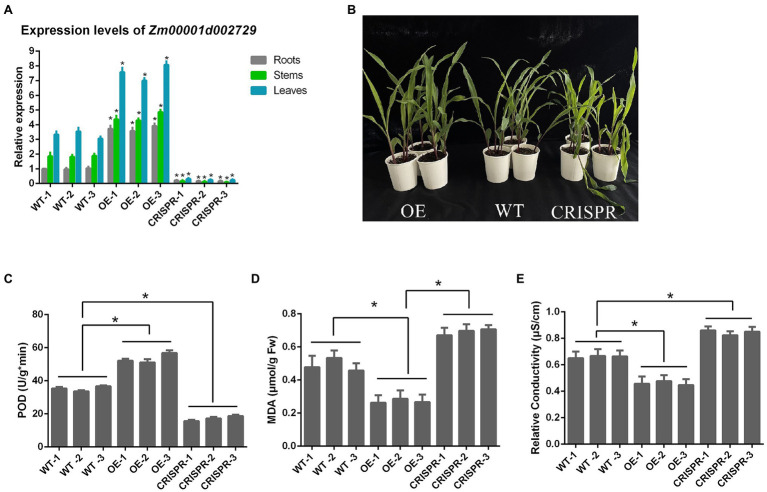
**(A)** Relative expression levels of *Zm00001d002729* in different maize tissues; **(B)** Comparison of maize morphology after exposure to 6°C cold stress for 24 h; **(C)** POD activity; **(D)** malondialdehyde (MDA) content; and **(E)** relative conductivity. *p < 0.05.

The relative expression levels of *Zm00001d002729* gene in the roots, stems, and leaves of each maize strain are shown in Figure 7. When the target gene was overexpressed, *Zm00001d002729* mRNA levels increased by an average of 274% in roots, 144% in stems, and 128% in leaves. After gene editing, the target gene dropped by an average of 82% in roots, 93% in stems, and 92% in leaves.

We also measured the POD activity, MDA content, and relative conductivity of these nine inbred maize lines after 24 h at 6°C (Figure 7). We found that in maize with overexpression of the target gene, the POD activity was significantly increased, the MDA content significantly decreased, and the relative conductivity was also significantly decreased (all *p* < 0.05); while in maize where the target gene was silenced, POD activity was decreased, and the MDA content and relative conductivity significantly increased. This indicates that *Zm00001d002729* is a positive regulator of cold resistance in maize. The higher the expression of *Zm00001d002729*, which could increase cold tolerance of maize.

## Discussion

### POD Activity and Maize Cold Resistance

In our study, we demonstrated that cold-tolerant maize had higher POD activity than cold-sensitive maize. The maize antioxidant stress pathway is a known important mechanism underlying cold tolerance (Özlem et al., 2013; Zhang et al., 2020; Yu et al., 2021). Most research on antioxidant enzymes has so far focused on maize catalase, which removes excess H_2_O_2_ from mitochondria and protects plants from cold stress (Prasad, 1997). Studies had shown that in the early stages of cold stress, POD activity in maize seedlings declines and then rises. With increased time and enhanced intensity of cold stress, POD activity has been shown to increased significantly (Guy et al., 1997; Roncatto and Pascholati, 1998; Šukalović et al., 2010; Ao et al., 2013). POD activity has been shown in previous studies to be higher in cold-tolerant than cold-sensitive varieties (Hodges et al., 1996; Wang et al., 2015), which is consistent with our results.

### QTLs Associated With Maize Cold Tolerance

The QTL we found to be associated with cold tolerance was on chromosome 3 in maize, located in bin 3.04. Fracheboud et al. (2002, 2004) studied the effect of low temperature on the photosynthetic mechanism of maize and analyzed five QTLs related to this function; one of these QTLs was located near the mmc0022 marker on chromosome 3, and explained 28% of the phenotypic variation, believed to be related to photosynthetic function at low temperatures in maize. The relevant gene is likely to be located on chromosome 3, close to the centromere; this is very close to bin 3.04. Interestingly, there are previous reports of other biotic or abiotic stress-related QTLs located near the QTL we identified as: Messmer et al. (2009) detected three drought-related stable QTLs located on chromosome 3 bin 3.04, and their contributions of phenotypic variation in this trait were 8.9, 10.8, and 6.3%. Balint-Kurti identified QTLs related to southern leaf blight resistance in two RIL populations (H99 × B73 and B73 × B52), also located in bin 3.04 (Balint-Kurti et al., 2008). Based on a genetic map of 246 polymorphic simple sequence repeat markers with an average genetic distance of 9.1 cM, Ding et al. (2008) used the compound interval mapping method (CIM) for QTL mapping to analyze associations with maize fusarium ear rot under four environments in a RIL population. Two QTLs, both located at bin 3.04 of chromosome 3, were detected in all environments, and these QTLs were located on either side of the markers umc1025 and umc1742, respectively; the QTL we identified in this study was very close to umc1742. Guan et al. (2012) found that a *Ragged leaves 1* (*Rg1*) maize mutant is susceptible to disease caused by abiotic stress, resulting in the extensive accumulation of H_2_O_2_ in diseased tissues and lesions on the leaves, sheaths, and bracts of maize. Interestingly, this is similar to the mechanism by which plants are subjected to cold stress (Weig et al., 1997). However, *Rg1* is located on chromosome 3 bin 3.04. In summary, for the QTL we discovered, there were almost no previous reports of association with maize cold tolerance traits, though there were some with tolerance to other stresses. This indicates that we have discovered a new QTL related to maize cold tolerance.

### Analysis of *Zm00001d002729* Associated With Maize Cold Tolerance

Functional annotation suggested that *Zm00001d002729* is an acyl-acyl carrier protein thioesterase. We found that overexpression of *Zm00001d002729* increases the cold tolerance of maize, while inhibiting the expression of this gene increases sensitivity to low temperatures. In previous studies, acyl-acyl carrier protein thioesterase is the key enzyme in the regulation of fatty acid carbon chain elongation during fatty acid synthesis. It can catalyze the hydrolysis of thioester bonds and terminate the synthesis of fatty acids. Its activity thus affects the content of various fatty acids in plants (Nieto et al., 2007). However, there are relatively few functional verifications of this activity in plant resistance to stress (Dormann et al., 2000; Wu et al., 2009). In a study by Raffaele et al. (2008), it was shown that mutation in acyl-acyl carrier protein thioesterase in *Arabidopsis thaliana* leads to serious defects in the fatty acid supply for long-chain fatty acid biosynthesis. *Arabidopsis thaliana* also lost its hypersensitive response to pathogen attack. Zhang et al. (2012) found that the overexpression of acyl-acyl carrier protein thioesterase in tobacco improves drought tolerance. The phase transition hypothesis of cold stress damage in plants states that when temperate plants suffer low temperature damage below a certain threshold, the biological membrane will first undergo a phase transformation of membrane lipids, from a liquid crystal phase to a gel phase. The fatty acid chains in the membrane lipids change from a disorderly to an orderly arrangement; the fluidity of the membrane is greatly reduced, and pores or cracks form in the membrane, increasing the permeability of the membrane and changing the structure of membrane-bound proteins. The efflux of a large amount of materials in the membrane destroys the ion balance between the inside and outside of the cell, leading to changes in physiological metabolism and functional disorders in plant cells (Lyons and Raison, 1970). We quoted the expression levels of *Zm00001d002729* from MaizeGDB (Supplementary File 13). We found that RNA-seq showed that the expression of target genes in V1 and V3 phases was extremely low in B73 (Winter et al., 2007; Makarevitch et al., 2015; Hoopes et al., 2019). In previous studies, it was found that B73 belong to cold-sensitive inbred line (Joets et al., 2018; Grzybowski et al., 2019; Jonczyk et al., 2020). Therefore, we speculated that the more cold-sensitive maize, the lower the expression levels of *Zm00001d002729*. It is consistent with the conclusions we got before. We do not yet understand the mechanism by which *Zm00001d002729* confers greater cold tolerance, and how this might fit with the phase transition hypothesis; however, our identification of the general function of this gene has laid the foundation for elucidating and exploiting this mechanism through genetic engineering in crops.

In conclusion, we successfully used GWAS and QTL mapping to successfully identify *Zm00001d002729*, a gene on maize chromosome 3 that is significantly related to POD activity. We used genetic transformation to overexpress or inhibit the expression of *Zm00001d002729* in maize, verifying its function as a cold tolerance gene. We have shown for the first time that *Zm00001d002729* positively regulates cold resistance in maize and may be a potentially useful tool to generate cold-resistant crops to ensure food security in the face of climate change.

## Data Availability Statement

The datasets presented in this study can be found in online repositories. The names of the repository/repositories and accession number(s) can be found at: https://www.ncbi.nlm.nih.gov/, PRJNA495031 and PRJNA763107.

## Author Contributions

YJ drafted the original manuscript. ZZ reviewed and edited the manuscript. YX and ZY conducted the experiments and performed the analysis. ZX, SG, and JQ performed the phenotyping. PW conducted formal analysis. RZ supervised the experiments. All authors contributed to the article and approved the submitted version.

## Funding

This research was supported by the Department of Science and Technology of Jilin Province (20200402026NC) and the National Key Research and Development Program of China (2019YFD1002603-1).

## Conflict of Interest

ZZ was employed by the company Novogene Co., Ltd.

The remaining authors declare that the research was conducted in the absence of any commercial or financial relationships that could be construed as a potential conflict of interest.

## Publisher’s Note

All claims expressed in this article are solely those of the authors and do not necessarily represent those of their affiliated organizations, or those of the publisher, the editors and the reviewers. Any product that may be evaluated in this article, or claim that may be made by its manufacturer, is not guaranteed or endorsed by the publisher.

## Footnotes

^1^ https://www.maizegdb.org/genome/assembly/Zm-B73-REFERENCE-GRAMENE-4.0

^2^ http://treesoft.sourceforge.net/

^3^ https://yanglab.westlake.edu.cn/software/gcta/#Overview

^4^ http://www.uniprot.org/

^5^ http://clovr.org/docs/clusters-of-orthologous-groups-cogs/

^6^ http://geneontology.org/

^7^ https://www.genome.jp/kegg/

^8^ https://www.ncbi.nlm.nih.gov/

^9^ http://crispr.hzau.edu.cn/CRISPR2/

^10^ http://www.csbio.sjtu.edu.cn/bioinf/Cell-PLoc-2/

## References

[ref1] Ao P. X. Li Z. G. Fan D. M. Gong M. (2013). Involvement of antioxidant defense system in chill hardening-induced chilling tolerance in *Jatropha curcas* seedlings. Acta Physiol. Plant. 35, 153–160. doi: 10.1007/s11738-012-1058-z

[ref2] Balint-Kurti P. J. Zwonitzer J. C. Pe M. E. Pea G. Lee M. Cardinal A. J. (2008). Identification of quantitative trait loci for resistance to southern leaf blight and days to anthesis in two maize recombinant inbred line populations. Phytopathology 98, 315–320. doi: 10.1094/PHYTO-98-3-0315, PMID: 18944082

[ref3] Barrett J. C. Fry B. Maller J. Daly M. J. (2005). Haploview: analysis and visualization of ld and haplotype maps. Bioinformatics 21, 263–265. doi: 10.1093/bioinformatics/bth457, PMID: 15297300

[ref4] Chou K. C. Shen H. B. (2008). Cell-PLoc: a package of web-servers for predicting subcellular localization of proteins in various organisms. Nat. Protoc. 3, 153–162. doi: 10.1038/nprot.2007.494, PMID: 18274516

[ref5] Ding J. Q. Wang X. M. Chander S. Yan J. B. Li J. S. (2008). Qtl mapping of resistance to fusarium ear rot using a ril population in maize. Mol. Breed. 22, 395–403. doi: 10.1007/s11032-008-9184-4

[ref6] Dormann P. Voelker T. A. Ohlrogge J. B. (2000). Accumulation of palmitate in *Arabidopsis* mediated by the acyl-acyl carrier protein thioesterase FATB1. Plant Physiol. 123, 637–644. doi: 10.1104/pp.123.2.637, PMID: 10859193 PMC59031

[ref7] Fracheboud Y. Jompuk C. Ribaut J. M. Stamp P. Leipner J. (2004). Genetic analysis of cold-tolerance of photosynthesis in maize. Plant Mol. Biol. 56, 241–253. doi: 10.1007/s11103-004-3353-6, PMID: 15604741

[ref8] Fracheboud Y. Ribaut J. M. Vargas M. Messmer R. Stamp P. (2002). Identification of quantitative trait loci for cold-tolerance of photosynthesis in maize (*Zea mays* L.). J. Exp. Bot. 53, 1967–1977. doi: 10.1093/jxb/erf040, PMID: 12177137

[ref9] Frame B. R. McMurray J. M. Fonger T. M. Main M. L. Taylor K. W. Torney F. J.. (2006). Improved agrobacterium-mediated transformation of three maize inbred lines using MS salts. Plant Cell Rep. 25, 1024–1034. doi: 10.1007/s00299-006-0145-2, PMID: 16710703

[ref10] Gao C. Jin H. Sheng Z. Zheng Y. Knapp A. (2009). Association of polyamines in governing the chilling sensitivity of maize genotypes. Plant Growth Regul. 57, 31–38. doi: 10.1007/s10725-008-9315-2

[ref11] Grzybowski M. Adamczyk J. Jonczyk M. Sobkowiak A. Szczepanik J. Frankiewicz K.. (2019). Increased photosensitivity at early growth as a possible mechanism of maize adaptation to cold springs. J. Exp. Bot. 70, 2887–2904. doi: 10.1093/jxb/erz096, PMID: 30825373 PMC6506767

[ref12] Guan H. Liu C. Zhao Y. Zeng B. Zhao H. Jiang Y.. (2012). Characterization, fine mapping and expression profiling of ragged leaves1 in maize. Theor. Appl. Genet. 125, 1125–1135. doi: 10.1007/s00122-012-1899-2, PMID: 22648613

[ref13] Guy C. Haskell D. Li Q. B. Zhang C. (1997). “Molecular chaperones: do they have a role in cold stress responses of plants?” in Plant Cold Hardiness. eds. Li P. H. Chen T. H. H. (Boston, MA: Springer), 109–129.

[ref14] Hodges D. M. Andrews C. J. Johnson D. A. Hamilton R. I. (1996). Antioxidant compound responses to chilling stress in differentially sensitive inbred maize lines. Physiol. Plant. 98, 685–692. doi: 10.2135/cropsci1997.0011183X003700030027x

[ref15] Hodges D. M. Andrews C. J. Johnson D. A. Hamilton R. I. (1997). Sensitivity of maize hybrids to chilling and their combining abilities at two developmental stages. Crop Sci. 37, 850–856. doi: 10.2135/cropsci1997.0011183X003700030026x

[ref16] Hoopes G. M. Hamilton J. P. Wood J. C. Esteban E. Pasha A. Vaillancourt B.. (2019). An updated gene atlas for maize reveals organ-specific and stress-induced genes. Plant J. 97, 1154–1167. doi: 10.1111/tpj.14184, PMID: 30537259 PMC6850026

[ref17] Hu S. Lübberstedt T. Zhao G. Lee M. (2016). QTL mapping of low-temperature germination ability in the maize IBM Syn4 RIL population. PLoS One 11:e0152795. doi: 10.1371/journal.pone.0152795, PMID: 27031623 PMC4816396

[ref18] Hund A. Fracheboud Y. Soldati A. Frascaroli E. Salvi S. Stamp P. (2004). QTL controlling root and shoot traits of maize seedlings under cold stress. Theor. Appl. Genet. 109, 618–629. doi: 10.1007/s00122-004-1665-1, PMID: 15179549

[ref19] Javadian N. Karimzadeh G. Mahfoozi S. Ghanati F. (2010). Cold-induced changes of enzymes, proline, carbohydrates, and chlorophyll in wheat. Russ. J. Plant Physiol. 57, 540–547. doi: 10.1134/S1021443710040126

[ref20] Jena K. K. Kim S. M. Suh J. P. Yang C. I. Kim Y. G. (2012). Identification of cold-tolerant breeding lines by quantitative trait loci associated with cold tolerance in rice. Crop Sci. 52, 517–523. doi: 10.2135/cropsci2010.12.0733

[ref21] Jia G. Huang X. Zhi H. Zhao Y. Zhao Q. Li W.. (2013). A haplotype map of genomic variations and genome-wide association studies of agronomic traits in foxtail millet (*Setaria italica*). Nat. Genet. 45, 957–961. doi: 10.1038/ng.2673, PMID: 23793027

[ref22] Jiang H. Li Y. Qin H. Li Y. Qi H. Li C.. (2018). Identification of major QTLs associated with first pod height and candidate gene mining in soybean. Front. Plant Sci. 9:1280. doi: 10.3389/fpls.2018.01280, PMID: 30283463 PMC6157441

[ref23] Joets J. Vitte C. Charcosset A. (2018). “Draft assembly of the F2 European maize genome sequence and its comparison to the B73 genome sequence: a characterization of genotype-specific regions,” in The Maize Genome. Compendium of Plant Genomes. eds. Bennetzen J. Flint-Garcia S. Hirsch C. Tuberosa R. (Springer International Publishing), 3–12.

[ref24] Jompuk C. Fracheboud Y. Stamp P. Leipner J. (2005). Mapping of quantitative trait loci associated with chilling tolerance in maize (*Zea mays* L.) seedlings grown under field conditions. J. Exp. Bot. 56, 1153–1163. doi: 10.1093/jxb/eri108, PMID: 15723825

[ref25] Jonczyk M. Sobkowiak A. Trzcinska-Danielewicz J. Sowinski P. (2020). Chromatin-level differences elucidate potential determinants of contrasting levels of cold sensitivity in maize lines. Plant Mol. Biol. Report. 39, 335–350. doi: 10.1007/s11105-020-01254-7

[ref26] Kaler A. S. Gillman J. D. Beissinger T. Purcell L. C. (2020). Comparing different statistical models and multiple testing corrections for association mapping in soybean and maize. Front. Plant Sci. 10:1794. doi: 10.3389/fpls.2019.01794, PMID: 32158452 PMC7052329

[ref27] Leipner J. Jompuk C. Camp K. H. Stamp P. Fracheboud Y. (2008). QTL studies reveal little relevance of chilling-related seedling traits for yield in maize. Theor. Appl. Genet. 116, 555–562. doi: 10.1007/s00122-007-0690-2, PMID: 18185918

[ref28] Li X. Wang G. Fu J. Li L. Jia G. Ren L.. (2018). QTL mapping in three connected populations reveals a set of consensus genomic regions for low temperature germination ability in *Zea mays* L. Front. Plant Sci. 9:65. doi: 10.3389/fpls.2018.00065, PMID: 29445387 PMC5797882

[ref29] Lu S. Zhang M. Zhang Z. Wang Z. Wu N. Song Y.. (2018). Screening and verification of genes associated with leaf angle and leaf orientation value in inbred maize lines. PLoS One 13:e0208386. doi: 10.1371/journal.pone.0208386, PMID: 30532152 PMC6285979

[ref30] Lyons J. M. Raison J. K. (1970). Oxidative activity of mitochondria isolated from plant tissues sensitive and resistant to chilling injury. Plant Physiol. 45, 386–389. doi: 10.1104/pp.45.4.386, PMID: 5427108 PMC396419

[ref31] Ma Y. H. Li S. Y. Lin H. Pan L. Y. Yang G. W. Lai Y. H.. (2018). Bioinformatics analysis of microarray data to reveal novel genes related to cold-resistance of maize. Russ. J. Plant Physiol. 65, 278–285. doi: 10.1134/S1021443718020152

[ref32] Ma X. Q. Tang J. H. Teng W. T. Yan J. B. Meng Y. J. Li J. S. (2007). Epistatic interaction is an important genetic basis of grain yield and its components in maize. Mol. Breed. 20, 41–51. doi: 10.2105/AJPH.2009.160853

[ref33] Makarevitch I. Waters A. J. West P. T. Stitzer M. Hirsch C. N. Ross-Ibarra J.. (2015). Transposable elements contribute to activation of maize genes in response to abiotic stress. PLoS Genet. 11:e1004915. doi: 10.1371/journal.pgen.1004915, PMID: 25569788 PMC4287451

[ref34] Meng L. Li H. Zhang L. Wang J. (2015). QTL IciMapping: integrated software for genetic linkage map construction and quantitative trait locus mapping in biparental populations. Crop J. 3, 269–283. doi: 10.1016/j.cj.2015.01.001

[ref35] Messmer R. Fracheboud Y. Banziger M. Vargas M. Stamp P. Ribaut J. M. (2009). Drought stress and tropical maize: qtl-by-environment interactions and stability of qtls across environments for yield components and secondary traits. Theor. Appl. Genet. 119, 913–930. doi: 10.1007/s00122-009-1099-x, PMID: 19597726

[ref36] Nieto C. Piron F. Dalmais M. Marco C. F. Moriones E. Gómez-Guillamón M. L.. (2007). EcoTILLING for the identification of allelic variants of melon *eIF4E*, a factor that controls virus susceptibility. BMC Plant Biol. 7, 1–9. doi: 10.1186/1471-2229-7-1, PMID: 17584936 PMC1914064

[ref37] Özlem D. İ. Körpe D. A. Sahin F. I. Haberal M. (2013). Hydrogen peroxide pretreatment of roots enhanced oxidative stress response of tomato under cold stress. Acta Physiol. Plant. 35, 1905–1913. doi: 10.1007/s11738-013-1228-7

[ref38] Pallant J. (2013). Spss survival manual: a step by step guide to data analysis using spss for windows. Aust. N. Z. J. Public Health 37, 597–598. doi: 10.1111/1753-6405.12166

[ref39] Pimentel C. Davey P. A. Juvik J. A. Long S. P. (2005). Gene loci in maize influencing susceptibility to chilling dependent photoinhibition of photosynthesis. Photosynth. Res. 85, 319–326. doi: 10.1007/s11120-005-5738-z, PMID: 16170634

[ref40] Prasad T. K. (1997). Role of catalase in inducing chilling tolerance in pre-emergent maize seedlings. Plant Physiol. 114, 1369–1376. doi: 10.1104/pp.114.4.1369, PMID: 12223775 PMC158429

[ref41] Raffaele S. Vailleau F. Léger A. Joubès J. Miersch O. Huard C.. (2008). A MYB transcription factor regulates very-long-chain fatty acid biosynthesis for activation of the hypersensitive cell death response in *Arabidopsis*. Plant Cell 20, 752–767. doi: 10.1105/tpc.107.054858, PMID: 18326828 PMC2329921

[ref42] Revilla P. Rodríguez V. M. Ordás A. Rincent R. Charcosset A. Giauffret C.. (2014). Cold tolerance in two large maize inbred panels adapted to european climates. Crop Sci. 54, 1981–1991. doi: 10.2135/cropsci2013.11.0733

[ref43] Rodríguez V. M. Butrón A. Malvar R. A. Ordás A. Revilla P. (2008). Quantitative trait loci for cold tolerance in the maize IBM population. Int. J. Plant Sci. 169, 551–556. doi: 10.1086/528755

[ref44] Rodríguez V. M. Butrón A. Rady M. O. A. Soengas P. Revilla P. (2014). Identification of quantitative trait loci involved in the response to cold stress in maize (*Zea mays* L.). Mol. Breed. 33, 363–371. doi: 10.1007/s11032-013-9955-4

[ref45] Roncatto M. C. Pascholati S. F. (1998). Change in activity and electrophoretic pattern of peroxidase in maize (Zea mays) and sorghum (*Sorghum bicolor*) leaves after treatment with yeast (*Saccharomyces cerevisiae*). Sci. Agric. 55, 395–402. doi: 10.1590/S0103-90161998000300007

[ref46] Shamimuzzaman M. Gardiner J. M. Walsh A. T. Triant D. A. Le Tourneau J. J. Tayal A.. (2020). MaizeMine: a data mining warehouse for the maize genetics and genomics database. Front. Plant Sci. 11:1630. doi: 10.3389/fpls.2020.592730, PMID: 33193550 PMC7642280

[ref47] Stam P. (1993). Construction of integrated genetic linkage maps by means of a new computer package: join map. Plant J. 3, 739–744. doi: 10.1111/j.1365-313X.1993.00739.x

[ref48] Strigens A. Freitag N. M. Gilbert X. Grieder C. Riedelsheimer C. Schrag T. A.. (2013). Association mapping for chilling tolerance in elite flint and dent maize inbred lines evaluated in growth chamber and field experiments. Plant Cell Environ. 36, 1871–1887. doi: 10.1111/pce.12096, PMID: 23488576

[ref49] Šukalović V. H. T. Veljović-Jovanović S. Maksimović J. D. Maksimović V. Pajić Z. (2010). Characterisation of phenol oxidase and peroxidase from maize silk. Plant Biol. 12, 406–413. doi: 10.1111/j.1438-8677.2009.00237.x, PMID: 20522176

[ref50] Trzcinska-Danielewicz J. Bilska A. Fronk J. Zielenkiewicz P. Sowiński P. (2009). Global analysis of gene expression in maize leaves treated with low temperature. i. Moderate chilling (14°C). Plant Sci. 177, 648–658. doi: 10.1016/j.plantsci.2009.09.001

[ref51] Wang S. Su S. Z. Wu Y. Li S. P. Shan X. H. Liu H. K.. (2015). Overexpression of maize chloride channel gene *ZmCLC-d* in *Arabidopsis thaliana* improved its stress resistance. Biol. Plant. 59, 55–64. doi: 10.1007/s10535-014-0468-8

[ref52] Wei B. Jing R. Wang C. Chen J. Mao X. Chang X.. (2009). Dreb1 genes in wheat (*Triticum aestivum* L.): development of functional markers and gene mapping based on SNPs. Mol. Breed. 23, 13–22. doi: 10.1007/s11032-008-9209-z

[ref53] Weig A. Deswarte C. Chrispeels M. J. (1997). The major intrinsic protein family of *Arabidopsis* has 23 members that form three distinct groups with functional aquaporins in each group. Plant Physiol. 114, 1347–1357. doi: 10.1104/pp.114.4.1347, PMID: 9276952 PMC158427

[ref54] Winter D. Vinegar B. Nahal H. Ammar R. Wilson G. V. Provart N. J. (2007). An “electronic fluorescent pictograph” browser for exploring and analyzing large-scale biological data sets. PLoS One 2:e718. doi: 10.1371/journal.pone.0000718, PMID: 17684564 PMC1934936

[ref55] Wu P. Z. Li J. Wei Q. Zeng L. Chen Y. P. Li M. R.. (2009). Cloning and functional characterization of an acyl-acyl carrier protein thioesterase (*JcFATB1*) from *Jatropha curcas*. Tree Physiol. 29, 1299–1305. doi: 10.1093/treephys/tpp054, PMID: 19671567

[ref56] Yan B. W. Xu X. X. Gu Y. N. Zhao Y. Zhao X. He L.. (2018). Genome-wide characterization and expression profiling of diacylglycerol acyltransferase genes from maize. Genome 61, 735–743. doi: 10.1139/gen-2018-0029, PMID: 30092654

[ref57] Yang J. Lee S. H. Goddard M. E. Visscher P. M. (2011). GCTA: a tool for genome-wide complex trait analysis. Am. J. Hum. Genet. 88, 76–82. doi: 10.1016/j.ajhg.2010.11.011, PMID: 21167468 PMC3014363

[ref58] Yang H. Wang K. (2015). Genomic variant annotation and prioritization with ANNOVAR and wANNOVAR. Nature 10, 1556–1566. doi: 10.1038/nprot.2015.105, PMID: 26379229 PMC4718734

[ref59] Yu T. Zhang J. Cao J. Cai Q. Li X. Sun Y.. (2021). Leaf transcriptomic response mediated by cold stress in two maize inbred lines with contrasting tolerance levels. Genomics 113, 782–794. doi: 10.1016/j.ygeno.2021.01.018, PMID: 33516847

[ref60] Zhang L. H. Jia B. Zhuo R. Y. Liu J. L. Pan H. Y. Baldwin T. C.. (2012). An acyl–acyl carrier protein thioesterase gene isolated from Wintersweet (*Chimonanthus praecox*), *CpFATB*, enhances drought tolerance in transgenic tobacco (*Nicotiana tobaccum*). Plant Mol. Biol. Report. 30, 433–442. doi: 10.1007/s11105-011-0359-5

[ref61] Zhang D. Li H. Wang J. Zhang H. Hu Z. Chu S.. (2016a). High-density genetic mapping identifies new major loci for tolerance to low-phosphorus stress in soybean. Front. Plant Sci. 7:372. doi: 10.3389/fpls.2016.00372, PMID: 27065041 PMC4811872

[ref62] Zhang Q. Liu Y. Yu Q. Ma Y. Gu W. Yang D. (2020). Physiological changes associated with enhanced cold resistance during maize (*Zea mays*) germination and seedling growth in response to exogenous calcium. Crop Pasture Sci. 71, 529–538. doi: 10.1071/CP19510

[ref63] Zhang Z. Shang H. Shi Y. Huang L. Li J. Ge Q.. (2016b). Construction of a high-density genetic map by specific locus amplified fragment sequencing (SLAF-seq) and its application to quantitative trait loci (QTL) analysis for boll weight in upland cotton (*Gossypium hirsutum*). BMC Plant Biol. 16, 79–18. doi: 10.1186/s12870-016-0741-4, PMID: 27067834 PMC4827241

[ref64] Zhao Z. Gu H. Sheng X. Yu H. Wang J. Huang L.. (2016). Genome-wide single-nucleotide polymorphisms discovery and high-density genetic map construction in cauliflower using specific-locus amplified fragment sequencing. Front. Plant Sci. 7:334. doi: 10.3389/fpls.2016.00334, PMID: 27047515 PMC4800193

[ref65] Zhao P. Zhou H. J. Potter D. Hu Y. H. Feng X. J. Dang M.. (2018). Population genetics, phylogenomics and hybrid speciation of *Juglans* in China determined from whole chloroplast genomes, transcriptomes, and genotyping-by-sequencing (GBS). Mol. Phylogenet. Evol. 126, 250–265. doi: 10.1016/j.ympev.2018.04.014, PMID: 29679714

[ref66] Zhou L. Wang S. B. Jian J. Geng Q. C. Wen J. Song Q.. (2015). Identification of domestication-related loci associated with flowering time and seed size in soybean with the RAD-seq genotyping method. Sci. Rep. 5, 1–8. doi: 10.1038/srep09350, PMID: 25797785 PMC4369735

