# Supplementary material for: Identification and Functional Verification of Cold Tolerance Genes in Spring Maize Seedlings Based on a Genome-Wide Association Study and Quantitative Trait Locus Mapping
Source: Front Plant Sci. 2021 Dec 9;12:776972. doi: 10.3389/fpls.2021.776972 (PMC8696014; doi:10.3389/fpls.2021.776972)
Supplement: Supplementary file 1 [file Data_Sheet_1.zip › Supplementary File 1.docx]

**Table S1.** Maize inbred lines used in the study.

| **Serial No.** | **Name** | **Pedigree** | **Origin** |
| --- | --- | --- | --- |
| W01 | Tie7922 | American hybrid P3382 | China |
| W02 | Shen5003 | American hybrid 3147 | China |
| W03 | HCL645 | NL9607/BE8066 | China |
| W04 | Si-4112 | A619/8112 | China |
| W05 | C8605-2 | Tie7922/Shen5003 | China |
| W06 | 7884 | Unknown | China |
| W07 | 7884-7 | 78-6/H84Ht | China |
| W08 | XL21 | (MNL947/ML6Q01-1)/MNL232 | China |
| W09 | H2671 | M4664/L7847 | China |
| W10 | Dan340 | Lv9/Podmaize | China |
| W11 | Dan598 | (Dan340/Danhuang11)/(Danhuang02/Dan599) | China |
| W12 | Longkang11 | Mo17/Zi330 | China |
| W13 | Huangzao4 | Tangsipingtou | China |
| W14 | H21 | Huangzao4/H84 | China |
| W15 | Mo17 | C103/187-2 | America |
| W16 | Zi330 | OH43/Keli67 | China |
| W17 | Ji1037 | (Mo17/Suwan1)/Mo17 | China |
| W18 | Dan1324 | Mo17/NN14D | China |
| W19 | Ji63 | Unknown | China |
| W20 | 4F1 | Mo17 mutant | China |
| W21 | 81162 | (Aijin25/Ye107)/106 | China |
| W22 | Shen137 | American hybrid 6JK111 | China |
| W23 | 8723 | Unknown | China |
| W24 | 835 | 718/8112 | China |
| W25 | Zhong128 | Zi7490/2118 | China |
| W26 | 9802 | Unknown | China |
| W27 | PH09B | PHP38/PHHB9 | America |
| W28 | Si-287 | 444/255 | China |
| W29 | Xi14 | Unknown | China |
| W30 | A801 | dan9042/(Dan9046/Mohuang9) | China |
| W31 | Dan988 | American hybrid PN78599 | China |
| W32 | Ji818 | VT157/Ji63 | China |
| W33 | Guma4404 | Unknown | China |
| W34 | Si-495 | (Mo17/L105)/Mo17 | China |
| W35 | D34 | Dan340 mutant | China |
| W36 | MD22 | Unknown | China |
| W37 | PHB1M | PH2KN/PH2KR | China |
| W38 | GS01 | Shen5003/Zi330 | China |
| W39 | GS02 | (Dan340/8902)/Dan360 | China |
| W40 | GS03 | Shen137E | China |
| W41 | GS04 | (FR600/8112)/8112 | China |
| W42 | WH8 | (628412/Mo17)/Mo17 | China |
| W43 | F8276 | Unknown | China |
| W44 | Km87 | American hybrid | China |
| W45 | Tong1643 | 7884-7Ht/Nan227 | China |
| W46 | Tong1922 | Tong635/(Mo17/Tie7922) | China |
| W47 | KX | German hybrid | China |
| W48 | D22 | (Ye478/Tie7922)/6314 | China |
| W49 | PH6WC | PH01N/PH09B | China |
| W50 | PH4CV | PH7V0/PHBE | China |
| W51 | Zheng58 | Ye478 mutant | China |
| W52 | Chang7-2 | Chinese hybrid Changdan2 | China |
| W53 | L201 | Shuye6-3/Tie7922 | China |
| W54 | L269 | German hybrid KX0769 | China |
| W55 | KWS10 | Unknown | Germany |
| W56 | KWS49 | Unknown | Germany |
| W57 | KW5G321 | Unknown | Germany |
| W58 | KW1A139 | Unknown | Germany |
| W59 | 9F592 | Unknown | Germany |
| W60 | S121 | (H201/Dan340)/H204 | China |
| W61 | M54 | X1132X/Tie7922 | China |
| W62 | K10 | (Chang3/Shen5003)/Chang3 | China |
| W63 | W9813 | Unknown | China |
| W64 | Ji853 | (Huangzao4/Zi330)/Zi330 | China |
| W65 | Ningchen07 | (78599/H21)/H21 | China |
| W66 | 666 | Unknown | China |
| W67 | T6039 | Unknown | China |
| W68 | F1113 | Unknown | China |
| W69 | P014 | Unknown | China |
| W70 | F1061 | Unknown | China |
| W71 | E1361 | Unknown | China |
| W72 | H299 | Unknown | China |
| W73 | 6F576 | Unknown | Germany |
| W74 | V76-1 | Unknown | China |
| W75 | T106 | (Ji853/Shen137)/Ji853 | China |
| W76 | Liao3180 | American hybrid PN3180 | China |
| W77 | 938401 | Unknown | Germany |
| W78 | 938402 | Unknown | Germany |
| W79 | M03 | X1132X/Zheng58 | China |
| W80 | M5972 | (Dan598/Chang7-2)/Chang7-2 | China |
