# Supplementary material for: Identification and Functional Verification of Cold Tolerance Genes in Spring Maize Seedlings Based on a Genome-Wide Association Study and Quantitative Trait Locus Mapping
Source: Front Plant Sci. 2021 Dec 9;12:776972. doi: 10.3389/fpls.2021.776972 (PMC8696014; doi:10.3389/fpls.2021.776972)
Supplement: Supplementary file 1 [file Data_Sheet_1.zip › Supplementary File 11.docx]

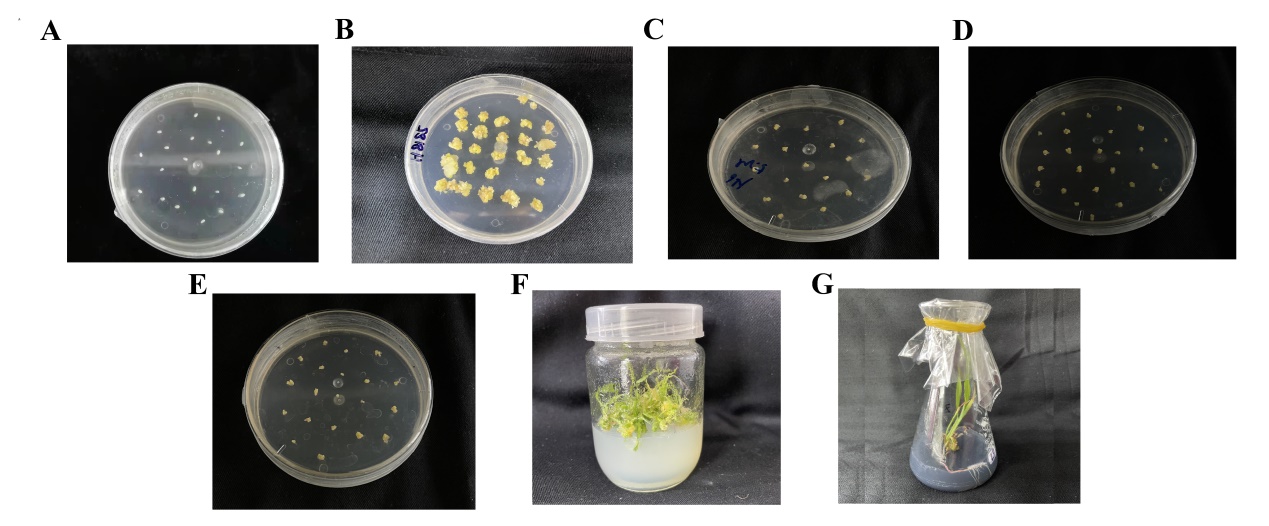


**Figure S11.** Tissue culture of maize. (A) Embryo culture; (B) subculture; (C) pre-culture; (D) co-culture; (E) screening culture; (F) elongation culture; (G) rooting culture.
