# Supplementary material for: Identification and Functional Verification of Cold Tolerance Genes in Spring Maize Seedlings Based on a Genome-Wide Association Study and Quantitative Trait Locus Mapping
Source: Front Plant Sci. 2021 Dec 9;12:776972. doi: 10.3389/fpls.2021.776972 (PMC8696014; doi:10.3389/fpls.2021.776972)
Supplement: Supplementary file 1 [file Data_Sheet_1.zip › Supplementary File 2.docx]

**Table S2**

| **Name of Genes** | **Primer Sequences** | **Use** |
| --- | --- | --- |
| *Zm00001d002729* F  *Zm00001d002729* R | AGGTGAAGCCTGTCCAAATC  GTTGGACGGAAGTCCTTGTT | qRT-PCR assay |
| *Actin-1* F  *Actin-1* R | CAAACAGAGAGAAAATGACGCAGA  CACCTGAATCCATCACAATACCA | Reference gene |
| *Zm00001d002729* F  *Zm00001d002729* R | GATCTGATGCAGCAGCAGATG  GGTCACCTTATGCAACCTTGC | Cloning |

List of primers used for the qRT-PCR and cloning of the target gene *Zm00001d002729*
