# Supplementary material for: Identification and Functional Verification of Cold Tolerance Genes in Spring Maize Seedlings Based on a Genome-Wide Association Study and Quantitative Trait Locus Mapping
Source: Front Plant Sci. 2021 Dec 9;12:776972. doi: 10.3389/fpls.2021.776972 (PMC8696014; doi:10.3389/fpls.2021.776972)
Supplement: Supplementary file 1 [file Data_Sheet_1.zip › Supplementary File 4.docx]

| **Category** | | **Number of SNPs** |
| --- | --- | --- |
| Total | | 24860241 |
| Upstream | | 554938 |
| Exonic | Missense | 0 |
|  | Stop gain | 9154 |
|  | Stop loss | 1223 |
|  | Synonymous | 308649 |
| Intronic | | 1342812 |
| Splicing | | 4680 |
| Downstream | | 545268 |
| Upstream/Downstream | | 28780 |
| Intergenic | | 21239263 |

**Table S4.** Single nucleotide polymorphism (SNP) statistics and functional annotation results.
