# Supplementary material for: Identification and Functional Verification of Cold Tolerance Genes in Spring Maize Seedlings Based on a Genome-Wide Association Study and Quantitative Trait Locus Mapping
Source: Front Plant Sci. 2021 Dec 9;12:776972. doi: 10.3389/fpls.2021.776972 (PMC8696014; doi:10.3389/fpls.2021.776972)
Supplement: Supplementary file 1 [file Data_Sheet_1.zip › Supplementary File 6.docx]

**Table S6.** Type of markers development.

| **Marker Type** | **W72♀ genotype** | **W10♂ genotype** | **Marker Number** | **Percentage** |
| --- | --- | --- | --- | --- |
| aa×bb | aa | bb | 1,847,696 | 32.08% |
| ab×cc | ab | cc | 0 | 0.00% |
| hk×hk | hk | hk | 1,045,227 | 18.15% |
| lm×ll | lm | ll | 1,362,360 | 23.65% |
| ab×cd | ab | cd | 0 | 0.00% |
| nn×np | nn | np | 1,504,585 | 26.12% |
| ef×eg | ef | eg | 0 | 0.00% |
| cc×ab | cc | ab | 0 | 0.00% |
| Total markers: | - | - | 5,759,868 | 100% |

**Note**: Marker Type: Parental genotype, such as ab × cc, ab and cc are the genotypes of the male parent and the female parent;

♀genotype: Maternal genotype;

♂genotype: Paternal genotype;

Marker number: The number of various types of markers;

Percentage: Percentage of each type of mark to the total number of valid marks;

Total: The total number of valid tag
