# Supplementary material for: Identification and Functional Verification of Cold Tolerance Genes in Spring Maize Seedlings Based on a Genome-Wide Association Study and Quantitative Trait Locus Mapping
Source: Front Plant Sci. 2021 Dec 9;12:776972. doi: 10.3389/fpls.2021.776972 (PMC8696014; doi:10.3389/fpls.2021.776972)
Supplement: Supplementary file 1 [file Data_Sheet_1.zip › Supplementary File 7.docx]

**Table S7.** Genetic linkage group statistics

| **Group** | **Marker number** | **Length (cM)** | **Average length (cM)** | **Max gap (cM)** | |
| --- | --- | --- | --- | --- | --- |
| Chr1 | 1163 | 1114.99 | 0.96 | | 10.14 |
| Chr2 | 795 | 610.98 | 0.77 | | 8.35 |
| Chr3 | 871 | 599.17 | 0.69 | | 6.63 |
| Chr4 | 1052 | 678.24 | 0.64 | | 8.12 |
| Chr5 | 619 | 414.49 | 0.67 | | 9.49 |
| Chr6 | 902 | 601.05 | 0.67 | | 10.7 |
| Chr7 | 754 | 756.15 | 1 | | 20.44 |
| Chr8 | 949 | 682.57 | 0.72 | | 10.55 |
| Chr9 | 643 | 613 | 0.95 | | 18.51 |
| Chr10 | 465 | 445.71 | 0.96 | | 15.35 |
| total | 8213 | 6516.35 | 0.79 | | 20.44 |
