# Supplementary material for: Identification and Functional Verification of Cold Tolerance Genes in Spring Maize Seedlings Based on a Genome-Wide Association Study and Quantitative Trait Locus Mapping
Source: Front Plant Sci. 2021 Dec 9;12:776972. doi: 10.3389/fpls.2021.776972 (PMC8696014; doi:10.3389/fpls.2021.776972)
Supplement: Supplementary file 1 [file Data_Sheet_1.zip › Supplementary File 8.docx]

**Table S8.** Genetic map showing the distribution and size of gaps.

| **Group** | **<5cM** | **5 to 10** | **10 to 20** | **>20cM** | **Ratio（%）** |
| --- | --- | --- | --- | --- | --- |
| Chr1 | 1139 | 22 | 2 | 0 | 97.94 |
| Chr2 | 786 | 9 | 0 | 0 | 98.87 |
| Chr3 | 865 | 6 | 0 | 0 | 99.31 |
| Chr4 | 1050 | 2 | 0 | 0 | 99.81 |
| Chr5 | 612 | 7 | 0 | 0 | 98.87 |
| Chr6 | 894 | 7 | 1 | 0 | 99.11 |
| Chr7 | 744 | 8 | 1 | 1 | 98.67 |
| Chr8 | 941 | 7 | 1 | 0 | 99.16 |
| Chr9 | 633 | 8 | 2 | 0 | 98.44 |
| Chr10 | 453 | 10 | 2 | 0 | 97.42 |
| Total | 8117 | 86 | 9 | 1 | 98.83 |
