# Supplementary figures and images for: Identification and Functional Verification of Cold Tolerance Genes in Spring Maize Seedlings Based on a Genome-Wide Association Study and Quantitative Trait Locus Mapping
Source: Front Plant Sci. 2021 Dec 9;12:776972. doi: 10.3389/fpls.2021.776972 (PMC8696014; doi:10.3389/fpls.2021.776972)

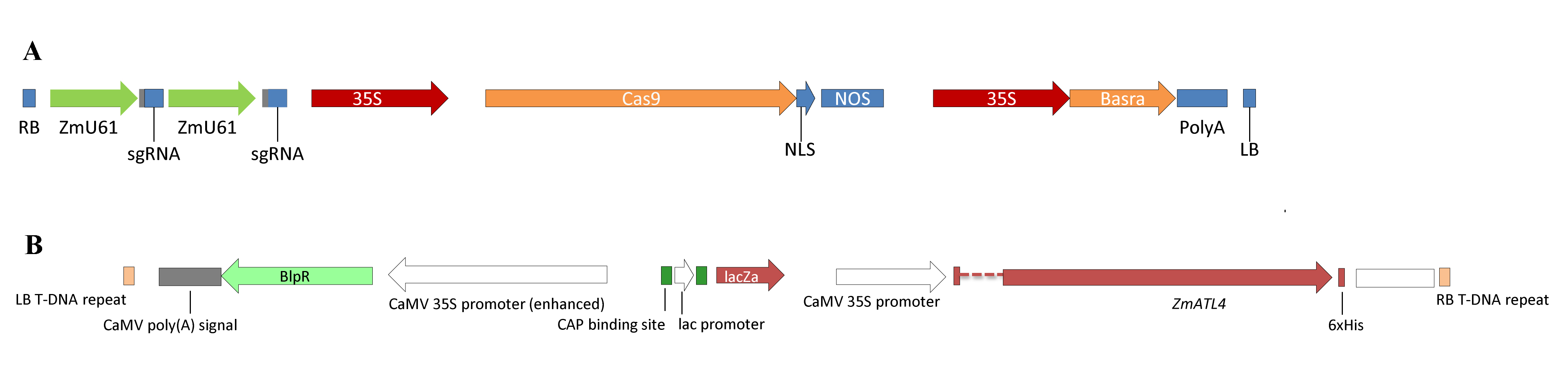


**Figure S10.** The structures of the *Zm00001d002729* overexpression vector and CRISPR/CAS9.

Supplement: Supplementary file 1 [file Data_Sheet_1.zip › Supplementary File 10.docx]

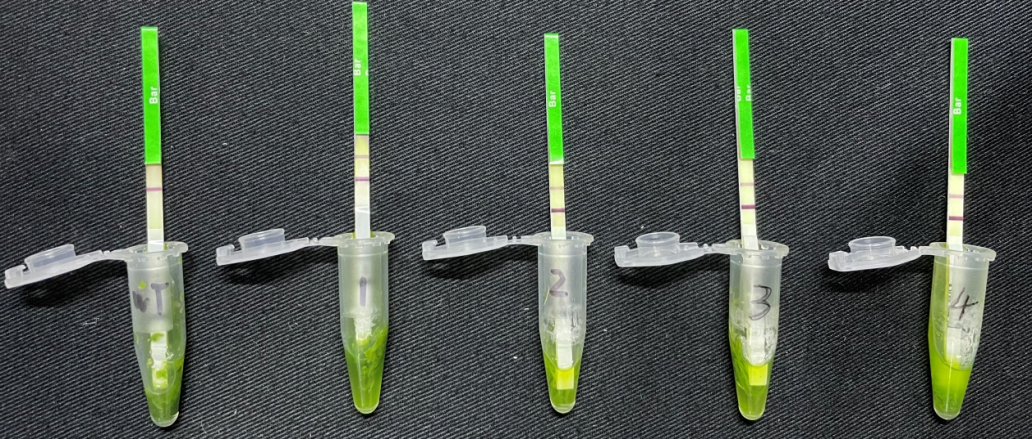


**Figure S12.** Test strips permitting the detection of genetically modified maize.

Supplement: Supplementary file 1 [file Data_Sheet_1.zip › Supplementary File 12.docx]

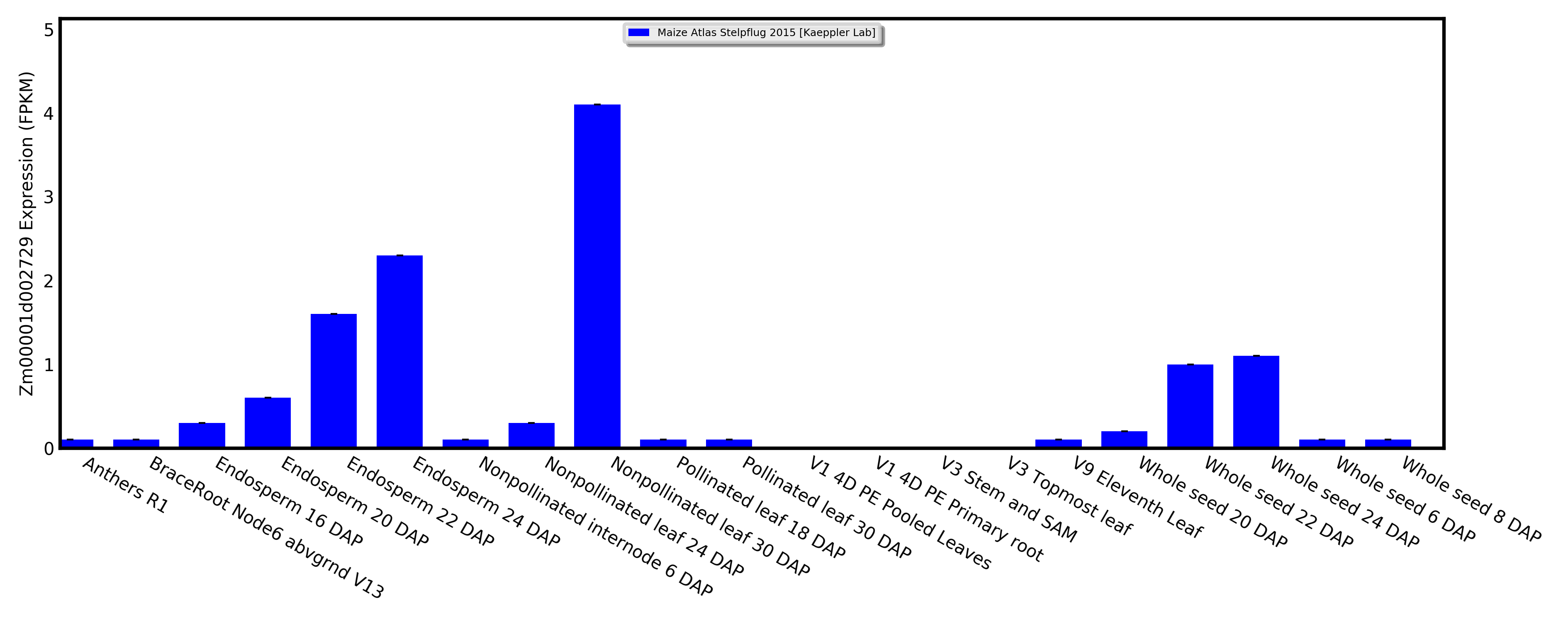

Supplement: Supplementary file 1 [file Data_Sheet_1.zip › Supplementary File 13.jpg]
