# Supplementary material for: Identification and Functional Verification of Cold Tolerance Genes in Spring Maize Seedlings Based on a Genome-Wide Association Study and Quantitative Trait Locus Mapping
Source: Front Plant Sci. 2021 Dec 9;12:776972. doi: 10.3389/fpls.2021.776972 (PMC8696014; doi:10.3389/fpls.2021.776972)
Supplement: Supplementary file 1 [file Data_Sheet_1.zip › Supplementary File 5.zip!/PC1-PC2-PC3 3D.pdf]

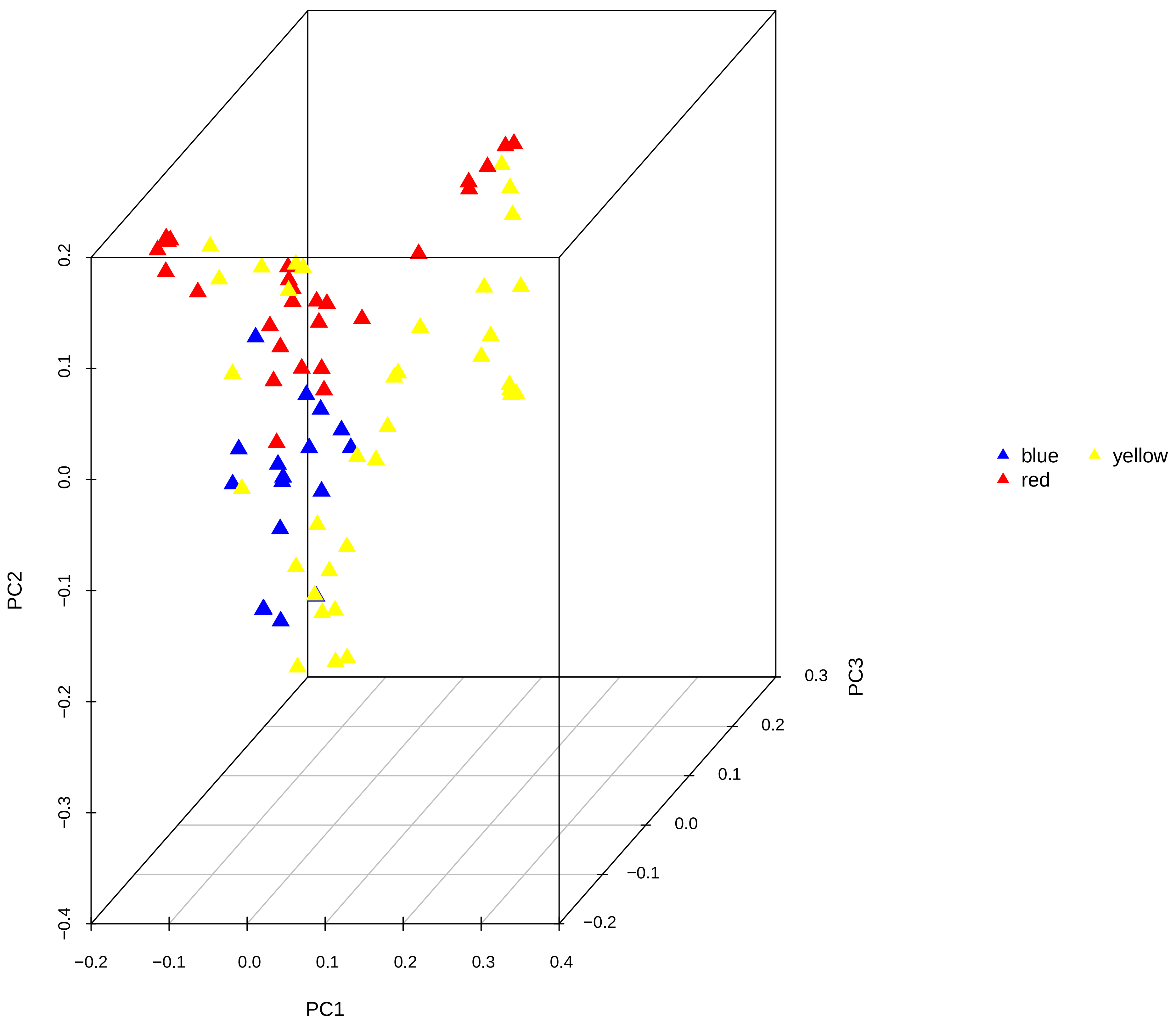

0.2
0.1
0.0
−0.1
−0.2
−0.3
−0.4
PC2
−0.2
−0.1
0.0
0.1
0.2
0.3
0.4
PC1
0.3
0.2
0.1
0.0
−0.1
−0.2
PC3
blue
red
yellow
