# Supplementary material for: Identification and Functional Verification of Cold Tolerance Genes in Spring Maize Seedlings Based on a Genome-Wide Association Study and Quantitative Trait Locus Mapping
Source: Front Plant Sci. 2021 Dec 9;12:776972. doi: 10.3389/fpls.2021.776972 (PMC8696014; doi:10.3389/fpls.2021.776972)
Supplement: Supplementary file 1 [file Data_Sheet_1.zip › Supplementary File 9.zip!/Colinearity.pdf]

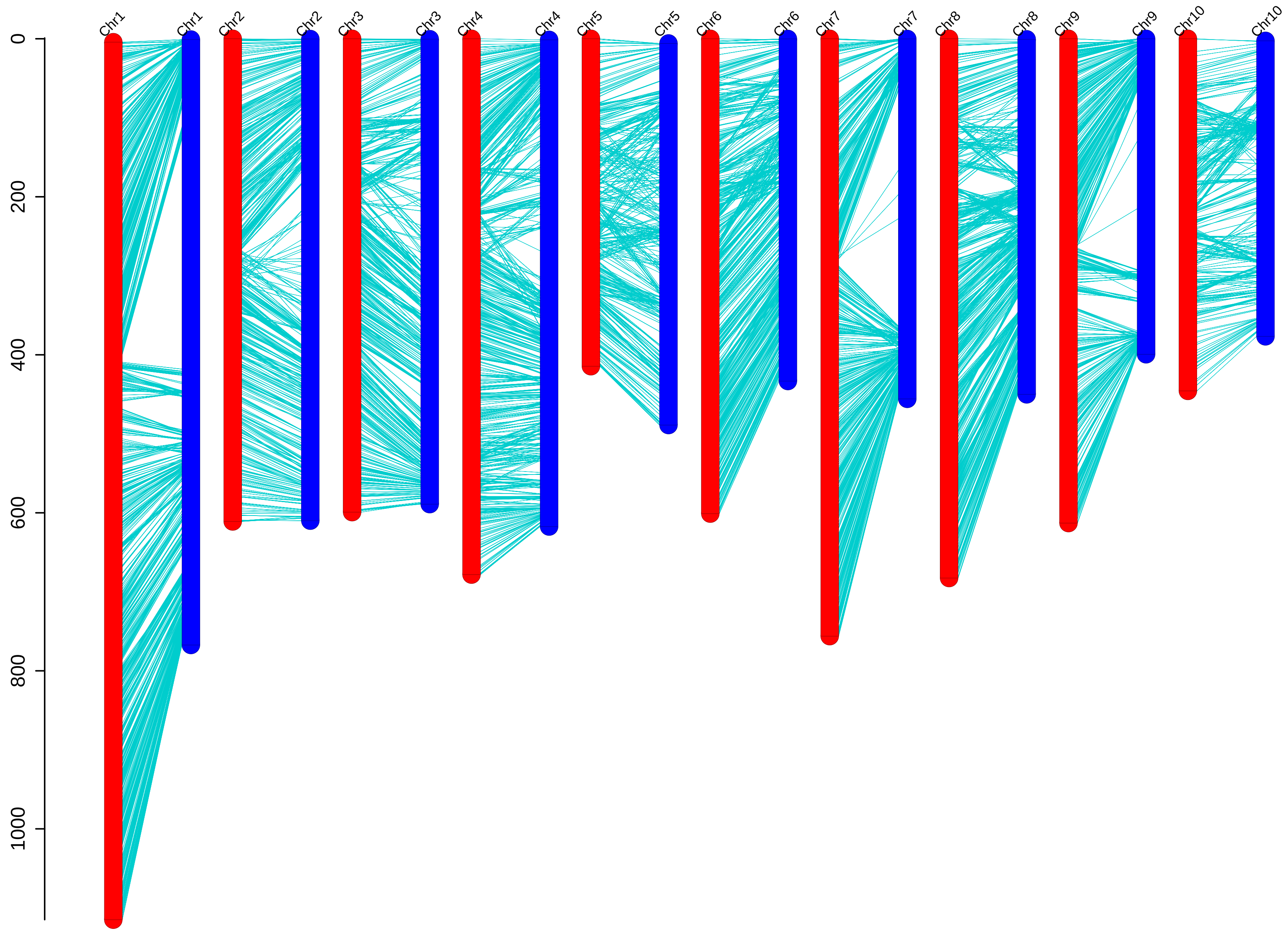

Chr1
Chr1
Chr2
Chr2
Chr3
Chr3
Chr4
Chr4
Chr5
Chr5
Chr6
Chr6
Chr7
Chr7
Chr8
Chr8
Chr9
Chr9
Chr10
Chr10
0
200
400
600
800
1000
